# A Novel Chromone Derivative with Anti-Inflammatory Property via Inhibition of ROS-Dependent Activation of TRAF6-ASK1-p38 Pathway

**DOI:** 10.1371/journal.pone.0037168

**Published:** 2012-06-15

**Authors:** Hailiang Liu, Rui Xu, Lili Feng, Wenjie Guo, Ning Cao, Cheng Qian, Peng Teng, Lu Wang, Xuefeng Wu, Yang Sun, Jianxin Li, Yan Shen, Qiang Xu

**Affiliations:** 1 State Key Laboratory of Pharmaceutical Biotechnology, School of Life Sciences, Nanjing University, Nanjing, China; 2 State Key Lab of Analytical Chemistry for Life Science, School of Chemistry and Chemical Engineering, Nanjing University, Nanjing, China; Albert Einstein College of Medicine, United States of America

## Abstract

The p38 MAPK signaling pathway plays a pivotal role in inflammation. Targeting p38 MAPK may be a potential strategy for the treatment of inflammatory diseases. In the present study, we show that a novel chromone derivative, DCO-6, significantly reduced lipopolysaccharide (LPS)-induced production of nitric oxide, IL-1β and IL-6, decreased the levels of iNOS, IL-1β and IL-6 mRNA expression in both RAW264.7 cells and mouse primary peritoneal macrophages, and inhibited LPS-induced activation of p38 MAPK but not of JNK, ERK. Moreover, DCO-6 specifically inhibited TLR4-dependent p38 activation without directly inhibiting its kinase activity. LPS-induced production of intracellular reactive oxygen species (ROS) was remarkably impaired by DCO-6, which disrupted the formation of the TRAF6-ASK1 complex. Administering DCO-6 significantly protected mice from LPS-induced septic shock in parallel with the inhibition of p38 activation and ROS production. Our results indicate that DCO-6 showed anti-inflammatory properties through inhibition of ROS-dependent activation of TRAF6-ASK1-p38 pathway. Blockade of the upstream events required for p38 MAPK action by DCO-6 may provide a new therapeutic option in the treatment of inflammatory diseases.

## Introduction

In mammals, the innate immune system is the first line of host defense against invading pathogens and is mediated by innate immune cells including macrophages [1]. Macrophages are critical effector cells contributing to the innate immune response against infection, as they are the most efficient pathogen scavengers and the predominant source of pro-inflammatory cytokines such as IL-1β, IL-6 and TNF-α, which are pivotal to promote inflammation at the site of infection and fight against pathogens [2], [3]. However, excess or inappropriate production of pro-inflammatory cytokines has serious consequences including tissue damage and septic shock [4], [5]. Therefore, inhibiting the synthesis or release of these cytokines and other cellular mediators emerges as a potential therapeutic approach for septic shock-like diseases associated with inappropriately amplified inflammatory responses.

The Toll-like receptor (TLR) family is one of the best-characterized pattern recognition receptor (PRR) families and is responsible for sensing invading pathogens. Recognition of bacterial components such as lipopolysaccharide (LPS) by TLRs results in the recruitment of multiple cytoplasmic signaling molecules involving MyD88, TRAF6, TIRAP and IRAK, which eventually activate downstream signaling components such as SAPK/JNK, p38 and NF-κB [6], [7], [8]. As an E3 ubiquitin ligase, TRAF6 interacts with various protein kinases including IRAK, SRC, and apoptosis signal-regulating kinase 1 (ASK1) and provides a link between distinct signaling pathways [9], [10], [11]. ASK1, a MAP kinase kinase kinase, plays an essential role in cytokine- and stress-induced apoptosis in mammalian cells by activating the MAP kinase kinase 4 (MKK4) and MKK3, which in turn lead to the activation of JNK and p38 pathways [12], [13], [14]. Recent studies have shown that ASK1 formed a complex with TRAF6 in response to LPS, and this complex formation and subsequent activation of p38 pathway required LPS-induced production of reactive oxygen species (ROS) [9], [10], [11].

Chromones have been extensively studied as bioactive compounds. They possess remarkable biological activities, including potent anti-inflammatory actions [15]. Our previous results revealed the diphenolic chromone derivatives as a new class of anti-inflammatory leads which showed potent inhibitory activity on nitric oxide (NO) production [16]. In this study, we investigated anti-inflammatory property of (E)-5,7-dihydroxy-3-(3-oxo-3-phenylprop-1-en-1-yl)-4H-chromen-4-one (DCO-6), a novel diphenolic chromone derivative. The results indicate that DCO-6 significantly reduced LPS-induced production of NO, IL-1β and IL-6 and decreased the levels of iNOS, IL-1β and IL-6 mRNA expression in macrophages. Moreover, LPS-induced activation of p38 MAPK was remarkably impaired by DCO-6 due to its inhibitory effects on the production of ROS and formation of TRAF6-ASK1 complex. In addition, DCO-6 alleviated LPS-induced mortality in murine model of septic shock, suggesting that DCO-6 may have use as a treatment for inflammatory diseases.

## Results

### DCO-6 inhibits LPS-induced NO, IL-1β and IL-6 Production in RAW264.7 and Peritoneal Macrophages

**Figure 1:**
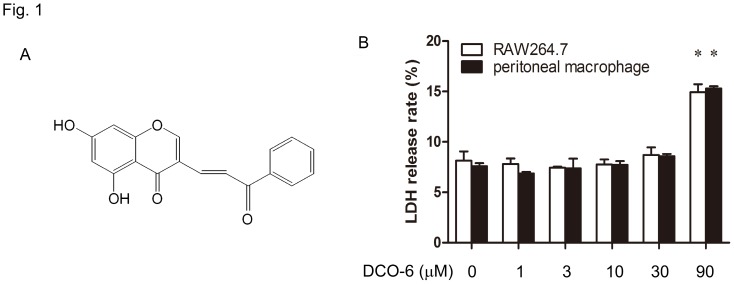
The effect of DCO-6 on cell viability of murine macrophages. (A) The chemical structure of DCO-6. (B) RAW264.7 cells or peritoneal macrophages from BALB/c mice were treated with various concentrations of DCO-6. After 24 h of incubation, LDH in the culture supernatant was tested. The absorbance value at 420 nm was measured by a microplate reader. The percentage of LDH released from the cells was determined using the formula: % release  =  LDH activity in supernatant/(LDH activity in supernatant + LDH activity in cell lysate). Data are shown as means ± S.D. of three independent experiments. *P<0.05 vs medium control.

The synthesis of (E)-5,7-dihydroxy-3-(3-oxo-3-phenylprop-1-en-1-yl)-4H-chromen-4- one (DCO-6) is outlined in supplementary material S1, and the ^1^H NMR spectrum of DCO-6 was shown in Fig. S1. DCO-6 at concentrations ranging from 1 to 30 µM failed to affect cell viability of both mouse RAW264.7 macrophages and primary mouse peritoneal macrophages (Fig. 1B and Fig. S2). Thus, up to 30 µM of DCO-6 was used in the following *in vitro* experiments.

**Figure 2:**
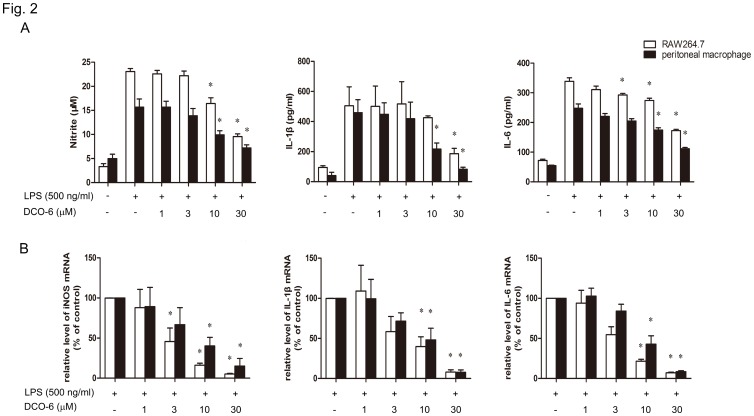
Inhibition of LPS-induced NO, IL-1β and IL-6 production by DCO-6 in murine macrophages. RAW264.7 cells or peritoneal macrophages from BALB/c mice were treated with various concentrations of DCO-6 in the absence or presence of LPS. (A) The levels of NO, IL-1β and IL-6 in the cell culture medium were determined 24 h after LPS stimulation as described in Methods. (B) The levels of iNOS, IL-1β and IL-6 mRNA were determined by real-time quantitative PCR 8 h after LPS stimulation. β-actin was used as an invariant control. Data are shown as means ± S.D. of three independent experiments. *P<0.05 vs LPS control.

To assess the effects of DCO-6 on LPS-induced production of cellular mediator in RAW264.7 and peritoneal macrophages, cell culture medium was harvested. Measuring nitrite as the index of NO production by the Griess method, we found that LPS treatment for 24 h resulted in a large amount of NO release in macrophages (Fig. 2A). Co-incubation of DCO-6 with LPS inhibited the formation of NO in a concentration-dependent manner. In addition, LPS-induced production of IL-1β and IL-6 was significantly reduced in macrophages treated with DCO-6 (Fig. 2A). When RNA was isolated and quantitative real-time PCR was performed to examine the effects of DCO-6 on gene expression, Co-incubation of DCO-6 with LPS also decreased the levels of iNOS, IL-1β and IL-6 mRNA expression (Fig. 2B), suggesting that NO, IL-1β and IL-6 reduction by DCO-6 may be related to transcriptional inhibition. However, neither TNF-α release nor mRNA induction was altered by DCO-6 at concentration of up to 30 µM (Fig. S3).

### DCO-6 Inhibits the Activation of p38 MAPK as well as NF-κB Signaling Induced by LPS in RAW264.7 Cells

**Figure 3:**
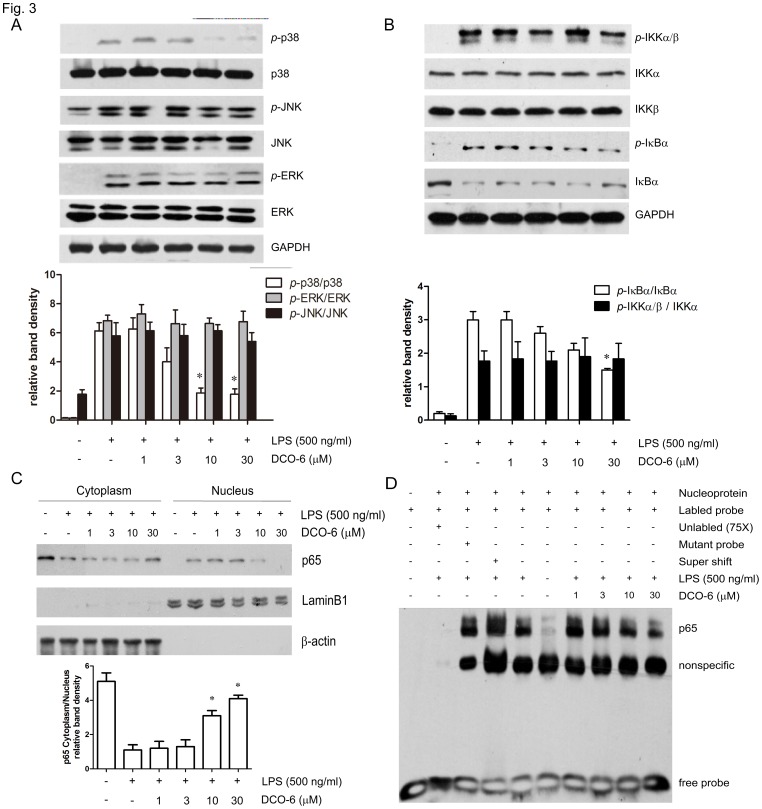
Effects of DCO-6 on the LPS-induced activation of MAPKs and NF-κB in RAW264.7 cells. Cells were treated with various concentrations of DCO-6 in the absence or presence of LPS for 6 h. (A–B) The protein levels of total and phosphorylated p38, JNK, ERK and IKKα, IKKβ, IκBα were determined at least three times, and the representative data are shown. (C) Cytoplasmic and nuclear proteins were extracted and assayed by immunoblotting analysis. Expressions of β-actin and LaminB1 were shown as loading controls. Bands from (A–C) were analyzed by densitometry. Quantitative data are shown. *P<0.05 vs LPS control. (D) Nuclear proteins were extracted and assayed by EMSA. A 75-fold excess of unlabelled oligonucleotide probe and mutant probe were used as controls.

In LPS signaling, activation of mitogen-activated protein kinases (MAPKs) and transcription factor NF-κB play essential roles in transcriptional induction of those genes involved in inflammation, such as iNOS, COX-2, TNF-α, IL-1β and IL-6 [17]. Here, we assessed the effects of DCO-6 on activation of MAPKs in RAW264.7 upon response to LPS. As shown in Fig. 3A, DCO-6 inhibited p38 MAPK phosphorylation induced by LPS in a concentration-dependent manner without any effect on total p38 MAPK expression. In contrast, DCO-6 did not affect phosphorylation of JNK, ERK induced by LPS. In spite of the failure in inhibition of phosphorylation of IKKα/β, DCO-6 slightly reduced phosphorylation of IκBα (Fig. 3B). Moreover, the nuclear localization of p65 subunit of NF-κB was inhibited by DCO-6, in line with the blockade of DNA binding activity of p65 in LPS-stimulated RAW264.7 cells (Fig. 3C and 3D).

### DCO-6 Inhibits p38 MAPK Activation Dependent on TLR Ligands in RAW264.7 Cells

**Figure 4:**
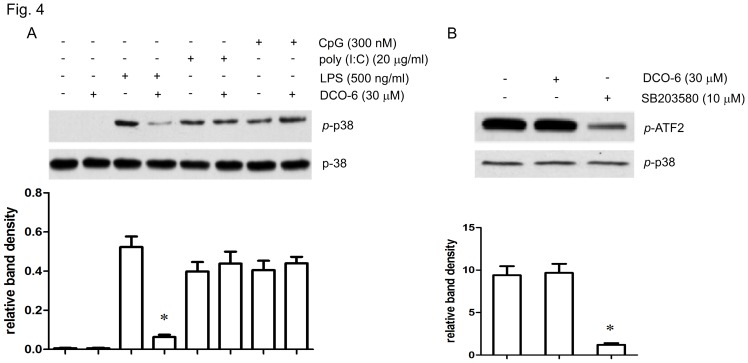
Effects of DCO-6 on p38 MAPK activation induced by different TLR ligands in RAW264.7 cells. (A) Cells were treated with DCO-6 in the absence or presence of indicated TLR ligands for 6 h. Whole cell lysates were prepared for Western blotting analysis. The protein levels of total and phosphorylated p38 were determined at least three times, and the representative data are shown. (B) Cells were stimulated by 500 ng/ml LPS for 6 h and the protein was collected. Phosphorylated p38 MAPK was immunoprecipitated. The immune complexes were used for testing the effects of DCO-6 and SB203580 on kinase activities. Representative data are shown. Bands from (A–B) were analyzed by densitometry. Quantitative data are shown. *P<0.05 vs control.

Next we used LPS (a ligand for TLR4), poly (I:C) (a synthetic double-stranded RNA and a ligand for TLR3) or unmethylated CpG (a ligand for TLR9) to stimulate RAW264.7 cells. As shown in Fig. 4A, p38 MAPK phosphorylation was substantially induced upon any stimuli. However, DCO-6 failed to reduce p38 phosphorylation induced by poly (I:C) or CpG, suggesting that DCO-6 specifically inhibited TLR4-dependent p38 activation. In an *in vitro* kinase assay to determine the direct effect of DCO-6 on the p38 MAPK, we found that, unlike p38 MAPK inhibitor SB203580, which can inhibit p38 MAPK at 10 µM, DCO-6 did not affect the kinase activity of p38 MAPK (Fig. 4B and Fig. S4).

### DCO-6 Inhibits the Production of Intracellular ROS and Formation of the TRAF6-ASK1 Complex in RAW264.7 Cells

**Figure 5:**
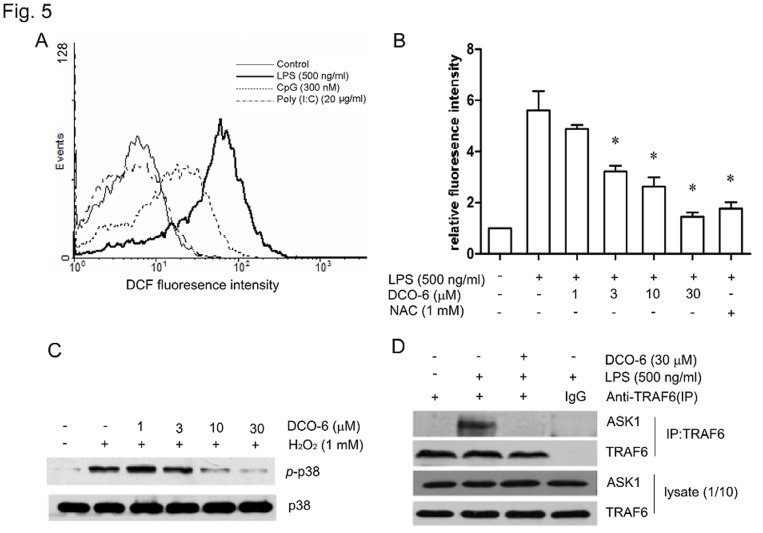
Effects of DCO-6 on the production of intracellular ROS and the formation of TRAF6-ASK1 complex in RAW264.7 cells. (A) Cells were incubated in the absence or presence of indicated TLR ligands for 6 h. Intracellular ROS production was detected by DCF fluorescence using flow cytometry. (B) Cells were treated with various concentrations of DCO-6 in the absence or presence of LPS. Intracellular ROS production was detected as mentioned above. Data are shown as means ± S.D. of three independent experiments. *P<0.05 vs LPS control. (C) Cells were treated with various concentrations of DCO-6 in the absence or presence of H_2_O_2_ for 6 h. Whole cell lysates were prepared for Western blotting analysis. The protein levels of total and phosphorylated p38 were determined at least three times, and representative data are shown. (D) The interaction between TRAF6 and ASK1 was measured by coimmunoprecipitation assay. Representative data are shown.

Given that the generation of ROS is required for TLR4-dependent activation of p38 but not JNK [18], we examined whether DCO-6 inhibited LPS-induced phosphorylation of p38 by reducing ROS production. After treatment for 6 h, LPS_,_ but not poly (I:C) or CpG, resulted in a large amount of ROS production in RAW264.7 cells (Fig. 5A). DCO-6 inhibited LPS-induced ROS production in a concentration-dependent manner (Fig. 5B) and 30 µM of DCO-6 showed the inhibitory activity comparable to 1 mM of the antioxidant N-acetyl-L-cystein (NAC). In addition, 1 mM of H_2_O_2_ in RAW264.7 cells induced p38 phosphorylation and Co-incubation of DCO-6 with H_2_O_2_ inhibited the phosphorylation of p38 in a concentration-dependent manner (Fig. 5C).

It is known that ROS mediates LPS-induced p38 activation by inducing the formation of the TRAF6-ASK1 complex [11]. Then, we examined the effects of DCO-6 on interactions between TRAF6 and ASK. As shown in Fig. 5D, TRAF6 did not coimmunoprecipitate ASK1 in the absence of LPS stimulation, whereas there was definite interaction between TRAF6 and ASK1 in RAW264.7 cells upon LPS stimulation. DCO-6 at 30 µM had no effect on the protein expression of TRAF6 and ASK1, but completely disrupted their interaction.

### DCO-6 Protects Mice from LPS-induced Septic Shock with a Significant Inhibition of p38 Activation

**Figure 6:**
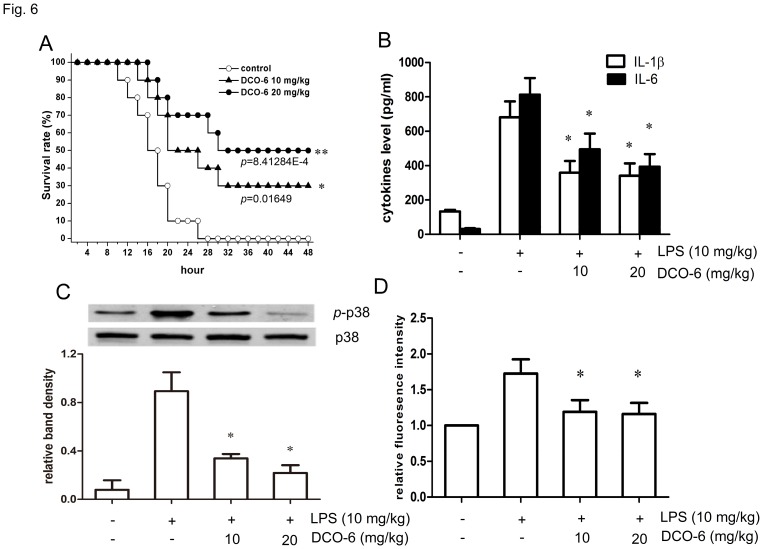
Protective effect of DCO-6 against LPS-induced septic shock in mice. BALB/c mice were administered LPS (10 mg/kg) and DCO-6 (10 or 20 mg/kg) or vehicle (olive oil) intraperitoneally. (A) Survival rate. (B) Serum cytokine levels of IL-1β and IL-6 were measured by ELISA 3 h after LPS injection. Data are shown as means ± S.D. of three independent experiments. n  = 10. *P<0.05 vs model control. (C) Peritoneal macrophages were isolated from the mice 3 h after LPS injection. Whole cell lysates were prepared, and the protein levels of total and phosphorylated p38 was detected by Western blotting analysis. Traces shown are representative of three independent experiments. Bands from (C) were analyzed by densitometry. Quantitative data are shown. *P<0.05 vs model control. (D) Peritoneal macrophages were isolated from the mice 3 h after LPS injection and incubated with DCFH-DA. DCF fluorescence distribution was detected by flow cytrometry. Data were analyzed by Cell Quest software. *P<0.05 vs model control.

To assess the protective effects of DCO-6 against LPS-induced lethal shock *in vivo*, BALB/c mice were intraperitoneally administered LPS (10 mg/kg) and DCO-6 (10 and 20 mg/kg). Cumulative proportions of mice surviving after lethal dose of LPS were shown in Fig. 6A. Single administration of DCO-6 improved survival in a dose-dependent manner. At 48 h after LPS injection, survival rate of DCO-6 treated mice (20 mg/kg) was 50% while none of untreated mice were alive. Moreover, LPS-induced IL-6 and IL-1β were significantly reduced in serum of DCO-6-treated groups (Fig. 6B). In addition, inhibition of p38 activation and ROS production was also observed in peritoneal macrophages from septic mice treated with DCO-6 (Fig. 6C and 6D).

## Discussion

This study examined the anti-inflammatory effect of the synthetic chromone derivative DCO-6 and its underlying mechanism. Our results suggest that the inhibition of TRAF6-ASK1-p38 signaling pathway by DCO-6 contributes to its anti-inflammatory action in reducing LPS-induced production of NO, IL-1β and IL-6 in macrophages and protecting against LPS-induced septic shock in mice. In contrast, DCO-6 did not affect the other two MAPK signaling cascades, ERK and JNK, elicited by LPS.

LPS-induced activation of p38 MAPK in macrophages has been widely demonstrated to correspond to the *in vitro* and *in vivo* effects of LPS on positive regulation of a variety of genes involved in inflammation. p38 MAPK signaling is implicated in LPS-induced transcriptional activation of pro-inflammatory genes, such as iNOS, COX-2, IL-1β and IL-6 [19], [20], [21], [22]. DCO-6 concentration-dependently inhibited LPS-induced p38 phosphorylation, an index of p38 MAPK activation. Accordingly, DCO-6 substantially decreased the levels of iNOS, IL-1β and IL-6 mRNA expression in macrophages. However, TNF-α production and mRNA induction was unaffected by DCO-6 *in vitro*. Multiple MAPK pathways activated by LPS are involved in the regulation of TNF-α expression [23]. It is possible that the p38 inhibition alone can not lead to significant attenuation of TNF-α transcriptional activity. Consistent with this finding, the selective p38 inhibitor SB203580 shows ineffectiveness on TNF-α promoter activity [24].

Despite the inhibition of p38 MAPK signaling induced by LPS, DCO-6 is not a direct inhibitor of p38 MAPK’s catalytic function as evidenced by the *in vitro* kinase assay, where DCO-6 did not affect the kinase activity of p38 MAPK. Upon stimulation by different TLR ligands, DCO-6 specifically inhibited TLR4-dependent p38 activation, suggesting that DCO-6 might block upstream events required for LPS-induced p38 MAPK activation. Indeed, we found that LPS_,_ but not poly (I:C) or CpG, resulted in a large amount of ROS production. And DCO-6 significantly inhibited ROS production in macrophages stimulated by LPS. Accumulating evidence has shown ROS are not only injurious by-products of cellular metabolism but also essential participants in cell signaling and regulation [25], [26], [27]. In LPS signaling, ROS can selectively mediate the formation of a complex between TRAF6 and the redox-sensitive ASK1, which in turn triggers p38 activation [11], [13]. In current study, DCO-6 completely disrupted the interaction between TRAF6 and ASK1 in RAW264.7 cells upon LPS stimulation, although the expression of TRAF6 and ASK1 protein was unaffected. These results indicate that DCO-6 may inhibit ROS-dependent activation of TRAF6 -ASK1-p38 pathway which leads to the production of inflammatory factors.

p38 MAPK’s role in the regulation of inflammatory cytokines and enzymes responsible for inflammation–like COX2, iNOS and MMPs–makes it an attractive drug target [28]. Several small molecular inhibitors targeting p38 have been developed and evaluated in animal models of inflammatory diseases [29], [30]. In this study, treatment with DCO-6 alleviated LPS-induced septic shock in mice and inhibited p38 activation *in vivo*. The anti-inflammatory potential of an oral p38 MAPK inhibitor was also evaluated in human endotoxemia [31]. Despite the strong rationale for MAPK inhibitors in human disease, direct proof of concept in the clinic has yet to be demonstrated, with most compounds demonstrating dose-limiting adverse effects [32]. Identification of new classes of compounds with greater specificity or a deeper knowledge of the other targets in p38 MAPK pathway could overcome these side-effects. Selective inhibition of more upstream events involved in p38 MAPK signaling may be a feasible strategy.

Although chromones and their structural analogues are known to play an important protective role against oxidation processes, many chromone-based compounds have demonstrated more fascinating properties. For example, it is reported that cromoglycate, a chromone complex, increases survival during an experimental model of sepsis by inhibiting HMGB1 release [33]. Unlike DCO-6, cromoglycate showed no effect on the LPS-induced p38 phosphorylation (Fig. S5). 30 µM of DCO-6 slightly, but significantly, reduced DNP-induced β-hexosaminidase release in Rat basophilic leukemia RBL-2H3 cells (Fig. S6). The results indicate that DCO-6 might have inhibitory effect on anaphylactic response through a different pathway from mast cell stabilizer cromoglycate. Recent study has shown that some chromone derivatives were synthesized and evaluated as p38 MAP kinase inhibitors [34]. However, the novel synthetic compound DCO-6 did not affect the kinase activity of p38 MAPK. DCO-6 showed low cytotoxicity and strong anti-inflammatory activity. Blocking the upstream events required for p38 MAPK action by DCO-6 may provide a new therapeutic option in the treatment of human inflammatory diseases.

## Materials and Methods

### Cells and Reagents

Murine RAW264.7 macrophages, obtained from the American Type Culture Collection (Rockville, MD), were cultured in DMEM (Invitrogen Corp., Carlsbad, CA) containing 5% fetal bovine serum (GIBCO, Grand Island, NY), 100 U/ml penicillin, and 100 µg/ml streptomycin in 5% CO_2_ at 37°C. Peritoneal macrophages elicited by thioglycollate broth (Sigma, St Louis, MO) were harvested by lavage of the peritoneal cavity. LPS from *Escherichia coli* (0111:B4), poly (I:C) and unmethylated CpG were purchased from Sigma, Invitrogen and Invitrogen, respectively. All other chemicals were obtained from Sigma.

### Mice

Male BALB/c, 6–8 weeks of age, were purchased from Experimental Animal Center of Jiangsu Province (Jiangsu, China). They were maintained with free access to pellet food and water in plastic cages at 21±2°C and kept on a 12 h light/dark cycle. Animal welfare and experimental procedures were carried out in accordance with the Guide for the Care and Use of Laboratory Animals (Ministry of Science and Technology of China, 2006) and the related ethical regulations of our university. All the animal experiments were approved by Nanjing University Animal Care and Use Committee (NJU-ACUC) and made to minimize suffering and to reduce the number of animals used.

### Measurement of Cytotoxicity, Nitrite and Cytokines

Cells (5×10^4^ per well) were cultured in a 96-well plate, and treated with various concentrations of DCO-6. After 24 h of incubation, LDH in the culture supernatant and in cell lysate were tested by using the CytoTox 96® Non-Radioactive Cytotoxicity Assay (Promega Corp., Shanghai). The percentage of LDH released from the cells was determined using the formula: % release  =  LDH activity in supernatant/(LDH activity in supernatant + LDH activity in cell lysate). For nitrite measurement, cells were treated with various concentrations of DCO-6 in the absence or presence of LPS (500 ng/ml) for 24 h, and then nitrite in the culture supernatant was measured by Griess reagent (Promega). And the nitrite concentration was calculated by using standard solution of sodium nitrite in the culture medium. For cytokine assay, IL-1β and IL-6 production were measured using ELISA kits from R&D systems (Minneapolis, MN) according to the manufacturer’s instructions.

### Real-time PCR

Real-time PCR was performed as described previously [35]. Briefly, RNA samples were treated by DNase and subjected to quantitative PCR, which was performed with the ABI Prism 7000 sequence detection system (Applied Biosystems, Foster City, CA) using SYBR Green I dye (Biotium, Inc.), and threshold cycle numbers were obtained using ABI Prism 7000 SDS software version 1.0. Conditions for amplification were 1 cycle of 94°C for 5 min followed by 40 cycles of 94°C for 30 s, 58°C for 30 s, and 72°C for 45 s. The primer sequences used in this study were as follows: iNOS forward, 5′-CAACATCAGGTCGGCCATCACT-3′; iNOS reverse, 5′-ACCAGAGGCAGCACATCAA AGC-3′; IL-1β forward, 5′-CTTCAGGCAGGCAGTATC ACTC-3′; IL-1β reverse, 5′-TGCAGTTGTCTAATGGGAACGT-3′; IL-6 forward, 5′-ACAACCACGGCCTTCC CTAC-3′; IL-6 reverse, 5′-TCTCATTTCCACGATT- TCCCAG-3′; β-actin: forward, 5′-TGCTGTCCCTGTATGCCTCT-3′; β-actin reverse, 5′-TTTGATGTCACGCACGATTT-3′.

### Western Blotting Analysis

Western blotting was performed as described previously [36]. Cells were collected and lysed in the lysis buffer containing Triton X-100. After 10,000 g centrifugation for 10 min, the protein content of the supernatant was determined by a BCA™ protein assay Kit (Pierce, Rochford, IL). The protein lysates were separated by 10% SDS-PAGE and subsequently electrotransferred onto a polyvinylidene diﬂuoride membrane (Millipore Corp., Bedford, MA). The membrane was blocked with 5% nonfat milk for 1 h at room temperature. The blocked membrane was incubated with the indicated antibodies. Protein bands were visualized using Western blotting detection system according to the manufacturer’s instructions.

For extraction of nucleoprotein, cells were collected and lysed in the lysis buffer (10 mM Hepes pH 7.9, 1.5 mM MgCl_2_, 10 mM KCl, 0.5 mM DTT, 2% NP-40, 1 mM PMSF) for 20 min, and the lysis buffer was centrifugated at 280 g for 10 minutes. the protein content of the supernatant was collected as cytoplasmic protein. Precipitation were washed twice and lysed in the lysis buffer containing Triton X-100 as nucleoprotein.

### p38 MAP Kinase Activity Assay

Cells were stimulated by 500 ng/ml LPS for 6 h and the protein was collected. p38 MAP Kinase activity was detected by p38 MAP Kinase Assay Kit (Cell Signaling Technology). Briefly, endogenous kinases were immuno- precipitated from cell lysates using phospho-p38 (Thr180/Tyr182) antibody bound to protein-A agarose. The beads were washed twice with lysis buffer and twice with kinase buffer (25 mM Hepes pH 7.4, 25 mM MgCl_2_, 25 mM β-glycerophosphate, 100 mM sodium orthovanadate, 2 mM DTT). The immunoprecipitates were incubated with DCO-6 (30 µM) or SB203580 (10 µM) for 10 min before the addition of kinase buffer containing ATP and ATF2 fusion protein as a substrate for p38. After further incubation for 30 min at 30°C, the phosphorylated ATF2 products were resolved by SDS-PAGE and analyzed by immunoblot according to the manufacturer’s instructions.

### Electrophoretic Mobility Shift assay (EMSA) Assay

EMSA was performed as described previously [37]. Brieﬂy, nucleoprotein (5 µg) was incubated in a 20 µl reaction volume (10 mM Tris–HCl, pH 7.8, 1 mM EDTA, 100 mM KCl, 5 mM MgCl_2_, 1 µg/ml poly dI.dC) for 20 min at 37°C and then loaded onto a 6% nondenaturing polyacrylamide gel. After cross-link transferred DNA to membrane, the bands were detected by chemiluminesecence. A 75-fold excess of unlabelled oligonucleotide probe and mutant probe were added in binding reaction to prove if the shifted bands binds specifically to NF-κB oligonucleotides. For supershift assays, 5 µg of rabbit anti-p65 polyclonal antibody (Cell Signaling Technology, Danvers, MA.) was incubated with the protein extract for 30 min prior to binding reaction.

### Measurement of Intracellular ROS

Cells (2×10^5^ per well) were cultured in a 6-well plate, and treated with various concentrations of DCO-6 in the presence of different TLR ligands for 6 h. Then the cells were harvested and incubated with 2,7-dichlorofluorescein diacetate (DCFH-DA, Invitrogen) at 37°C for 20 min and washed twice with cold PBS. DCF fluorescence distribution was detected by flow cytrometry on a FACScan (Becton Dickinson) at an excitation wavelength of 488 nm and an emission wavelength of 525 nm. Data were analyzed by Cell Quest software (Molecular Devices Corporation).

### Coimmunoprecipitation Assay

For coimmunoprecipitation, cells (1×10^8^) were lysed in lysis buffer containing Triton X-100 and cell lysates were immunoprecipitated with polyclonal mouse antibody to TRAF6 or control mouse immunoglobulin G with protein A/G–Sepharose. The beads were washed, separated by SDS-PAGE and analyzed by immunoblot with antibodies to ASK1 and TRAF6 (Santa Cruz). Protein bands were visualized using Western blotting detection system.

### LPS-induced Septic Shock in Mice

BALB/c mice were administered LPS at 10 mg/kg intraperitoneally and survival was monitored for 48 h (n  = 10 per group). DCO-6 (10 or 20 mg/kg) or vehicle (olive oil) was administered intraperitoneally at the day of LPS injection. In some experiments, blood samples were collected 3 h post LPS, and serum levels of IL-1β and IL-6 were measured using ELISA kit from R&D systems according to the manufacturer’s instructions.

### Statistical Analysis

All data represent as means ± S.D. of three independent experiments. Statistical analyses were performed using one-way analysis of variance (ANOVA), followed by Student two-tailed t test. Mortality differences between groups were evaluated by the Kaplan-Meier method. P <0.05 was considered significant.

## Supporting Information

Figure S1 **The ^1^H NMR spectrum of DCO-6.**
(TIF)

Figure S2 **The effect of DCO-6 on cell viability of murine macrophages.** RAW264.7 cells or peritoneal macrophages from BALB/c mice were treated with various concentrations of DCO-6. After 24 h of incubation, the cell viability was assessed by MTT assay. Data are shown as means ± S.D. of three independent experiments. ^*^P<0.05 vs medium control.
(TIF)

Figure S3 **The effect of DCO-6 on TNF-α production in murine macrophages.** RAW264.7 cells or peritoneal macrophages from BALB/c mice were treated with various concentrations of DCO-6 in the absence or presence of LPS. (A) The levels of TNF-α in the cell culture medium were determined 24 h after LPS stimulation. (B) The levels of TNF-α mRNA were determined by real-time quantitative PCR 8 h after LPS stimulation. β-actin was used as an invariant control. Data are shown as means ± S.D. of three independent experiments.
(TIF)

Figure S4 **Effects of DCO-6 on LPS-induced p38 MAPK activation in RAW264.7** **cells.** Cells were treated with DCO-6 and SB203580 in the absence or presence of LPS for 6 h. Endogenous kinases were immunoprecipitated from cell lysates using phospho-p38 (Thr180/Tyr182) antibody bound to protein-A agarose. The phosphorylated *p*-p38 products were analyzed by immunoblot. Representative data are shown.
(TIF)

Figure S5 **Effects of cromoglycate on p38 MAPK activation induced by LPS in RAW264.7** **cells.** Cells were treated with various concentrations of cromoglycate in the absence or presence of LPS for 6 h. Whole cell lysates were prepared for Western blotting analysis. The phosphorylated p38 level was analyzed by immunoblot.
(TIF)

Figure S6 **DCO-6 inhibited DNP-BSA-induced β-hexosaminidase release in RBL-2H3 cells.** As described in supplementary material S1, cells were seeded in 96-well plates (3×10^4^ cells/well) with or without 0.45 µg/ml anti-dinitrophenyl (DNP) IgE. After overnight incubation, the sensitized cells were treated with the indicated concentration of DCO-6 for 24 h. DNP-BSA (1 µg/ml) were added for 30 min and β-hexosaminidase release levels were detected. ^*^P<0.05 vs DNP-BSA control.
(TIF)

Supplementary Materials S1 (DOC)

## References

[1] Akira S, Uematsu S, Takeuchi O (2006). Pathogen Recognition and Innate Immunity.. Cell.

[2] Underhill DM, Ozinsky A (2002). Phagocytosis of microbes: complexity in action.. Annu Rev Immunol.

[3] Li W, Ashok M, Li J, Yang H, Sama AE (2007). A major ingredient of green tea rescues mice from lethal sepsis partly by inhibiting HMGB1.. PLoS One.

[4] Takeuchi O, Akira S (2010). Pattern recognition receptors and inflammation.. Cell.

[5] Cohen J (2002). The immunopathogenesis of sepsis.. Nature.

[6] Medzhitov R (2001). Toll-like receptors and innate immunity.. Nat Rev Immunol.

[7] Schnare M, Barton GM, Holt AC, Takeda K, Akira S (2001). Toll-like receptors control activation of adaptive immune responses.. Nat Immunol.

[8] Hoffmann JA, Reichhart JM (2002). Drosophila innate immunity: an evolutionary perspective.. Nat Immunol.

[9] Takatsuna H, Kato H, Gohda J, Akiyama T, Moriya A (2003). Identification of TIFA as an adapter protein that links tumor necrosis factor receptor-associated factor 6 (TRAF6) to interleukin-1 (IL-1) receptor-associated kinase-1 (IRAK-1) in IL-1 receptor signaling.. Journal of Biological Chemistry.

[10] Funakoshi-Tago M, Tago K, Sonoda Y, Tominaga S, Kasahara T (2003). TRAF6 and C-SRC induce synergistic AP-1 activation via PI3-kinase-AKT-JNK pathway.. Eur J Biochem.

[11] Matsuzawa A, Saegusa K, Noguchi T, Sadamitsu C, Nishitoh H (2005). ROS-dependent activation of the TRAF6-ASK1-p38 pathway is selectively required for TLR4-mediated innate immunity.. Nat Immunol.

[12] Nishitoh H, Matsuzawa A, Tobiume K, Saegusa K, Takeda K (2002). ASK1 is essential for endoplasmic reticulum stress-induced neuronal cell death triggered by expanded polyglutamine repeats.. Genes Dev.

[13] Ichijo H, Nishida E, Irie K, ten Dijke P, Saitoh M (1997). Induction of apoptosis by ASK1, a mammalian MAPKKK that activates SAPK/JNK and p38 signaling pathways.. Science.

[14] Matsuzawa A, Ichijo H (2001). Molecular mechanisms of the decision between life and death: regulation of apoptosis by apoptosis signal-regulating kinase 1.. J Biochem.

[15] Sharma SK, Kumar S, Chand K, Kathuria A, Gupta A (2011). An update on natural occurrence and biological activity of chromones.. Curr Med Chem.

[16] Liu GB, Xu JL, Geng M, Xu R, Hui RR (2010). Synthesis of a novel series of diphenolic chromone derivatives as inhibitors of NO production in LPS-activated RAW264.7 macrophages.. Bioorg Med Chem.

[17] Leon CG, Tory R, Jia J, Sivak O, Wasan KM (2008). Discovery and development of toll-like receptor 4 (TLR4) antagonists: a new paradigm for treating sepsis and other diseases.. Pharm Res.

[18] Hsu HY, Wen MH (2002). Lipopolysaccharide-mediated reactive oxygen species and signal transduction in the regulation of interleukin-1 gene expression.. J Biol Chem.

[19] Kristof AS, Marks-Konczalik J, Moss J (2001). Mitogen-activated protein kinases mediate activator protein-1-dependent human inducible nitric-oxide synthase promoter activation.. J Biol Chem.

[20] Guan Z, Buckman SY, Pentland AP, Templeton DJ, Morrison AR (1998). Induction of cyclooxygenase-2 by the activated MEKK1–> SEK1/MKK4–>p38 mitogen-activated protein kinase pathway.. J Biol Chem.

[21] Kirkwood KL, Rossa C (2009). The potential of p38 MAPK inhibitors to modulate periodontal infections.. Curr Drug Metab.

[22] Dai JN, Zong Y, Zhong LM, Li YM, Zhang W (2011). Gastrodin inhibits expression of inducible NO synthase, cyclooxygenase-2 and proinflammatory cytokines in cultured LPS-stimulated microglia via MAPK pathways.. PLoS One.

[23] Zhu W, Downey JS, Gu J, Di Padova F, Gram H (2000). Regulation of TNF expression by multiple mitogen-activated protein kinase pathways.. J Immunol.

[24] Ho FM, Lai CC, Huang LJ, Kuo TC, Chao CM (2004). The anti-inflammatory carbazole, LCY-2-CHO, inhibits lipopolysaccharide-induced inflammatory mediator expression through inhibition of the p38 mitogen-activated protein kinase signaling pathway in macrophages.. Br J Pharmacol.

[25] Thannickal VJ, Fanburg BL (2000). Reactive oxygen species in cell signaling.. American Journal of Physiology-Lung Cellular and Molecular Physiology.

[26] Kamata H, Hirata H (1999). Redox regulation of cellular signalling.. Cellular Signalling.

[27] Haschemi A, Chin BY, Jeitler M, Esterbauer H, Wagner O (2011). Carbon monoxide induced PPARgamma SUMOylation and UCP2 block inflammatory gene expression in macrophages.. PLoS One.

[28] Schindler JF, Monahan JB, Smith WG (2007). p38 pathway kinases as anti-inflammatory drug targets.. J Dent Res.

[29] Underwood DC, Osborn RR, Kotzer CJ, Adams JL, Lee JC (2000). SB 239063, a potent p38 MAP kinase inhibitor, reduces inflammatory cytokine production, airways eosinophil infiltration, and persistence.. J Pharmacol Exp Ther.

[30] Badger AM, Bradbeer JN, Votta B, Lee JC, Adams JL (1996). Pharmacological profile of SB 203580, a selective inhibitor of cytokine suppressive binding protein/p38 kinase, in animal models of arthritis, bone resorption, endotoxin shock and immune function.. J Pharmacol Exp Ther.

[31] Branger J, van den Blink B, Weijer S, Madwed J, Bos CL (2002). Anti-inflammatory effects of a p38 mitogen-activated protein kinase inhibitor during human endotoxemia.. J Immunol.

[32] Cohen S, Fleischmann R (2010). Kinase inhibitors: a new approach to rheumatoid arthritis treatment.. Curr Opin Rheumatol.

[33] Ramos L, Pena G, Cai B, Deitch EA, Ulloa L (2010). Mast cell stabilization improves survival by preventing apoptosis in sepsis.. J Immunol.

[34] Dyrager C, Mollers LN, Kjall LK, Alao JP, Diner P (2011). Design, Synthesis, and Biological Evaluation of Chromone-Based p38 MAP Kinase Inhibitors..

[35] Wang L, Shen Y, Song R, Sun Y, Xu J (2009). An anticancer effect of curcumin mediated by down-regulating phosphatase of regenerating liver-3 expression on highly metastatic melanoma cells.. Mol Pharmacol.

[36] Song R, Qian F, Li YP, Sheng X, Cao SX (2009). Phosphatase of regenerating liver-3 localizes to cyto-membrane and is required for B16F1 melanoma cell metastasis in vitro and in vivo.. PLoS One.

[37] Xu J, Cao S, Wang L, Xu R, Chen G (2011). VEGF promotes the transcription of the human PRL-3 gene in HUVEC through transcription factor MEF2C.. PLoS One.

