# Supplementary figures and images for: A Novel Chromone Derivative with Anti-Inflammatory Property via Inhibition of ROS-Dependent Activation of TRAF6-ASK1-p38 Pathway
Source: PLoS One. 2012 Jun 15;7(8):e37168. doi: 10.1371/journal.pone.0037168 (PMC3376149; doi:10.1371/journal.pone.0037168)

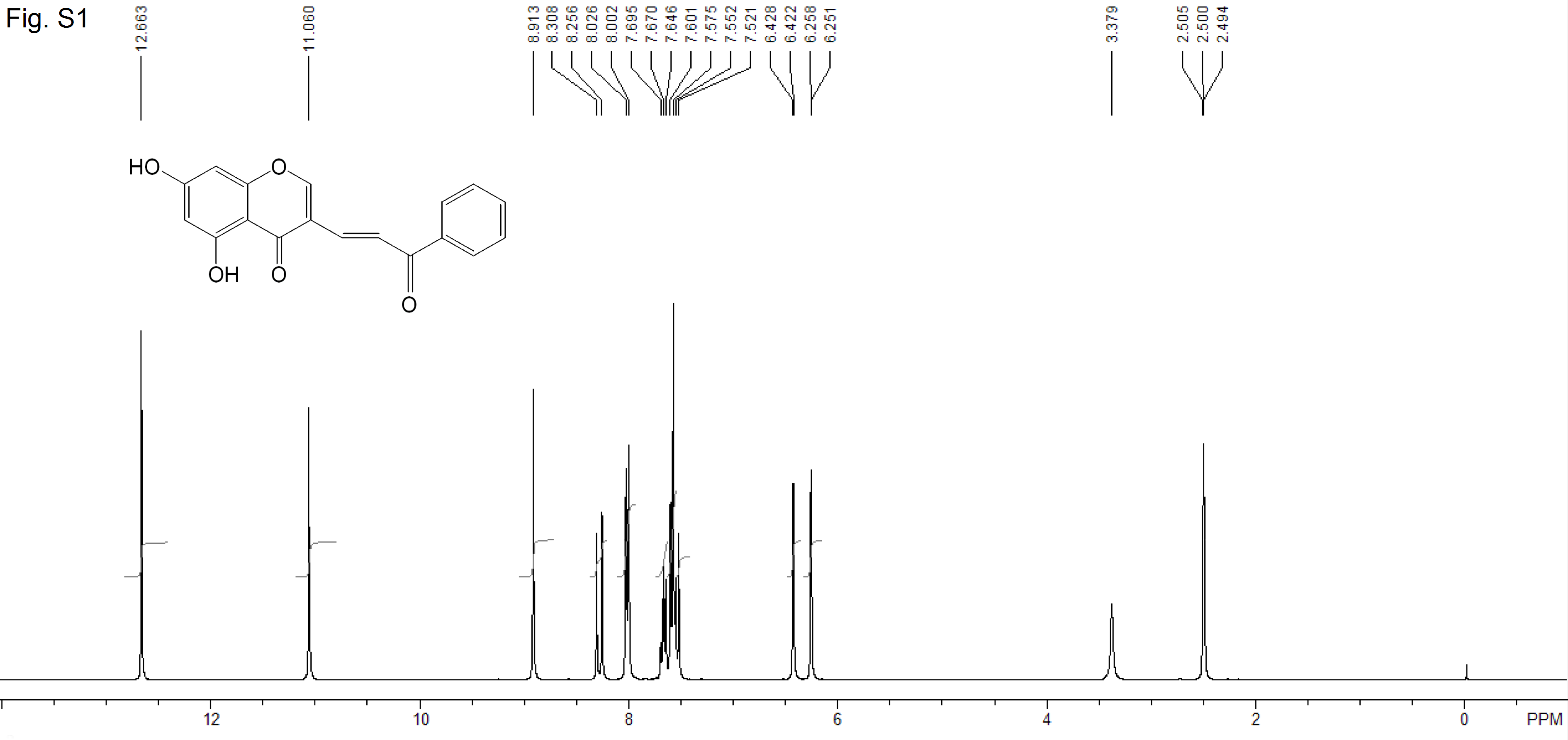

Supplement: Figure S1 — The 1H NMR spectrum of DCO-6. (TIF) [file pone.0037168.s001.tif]

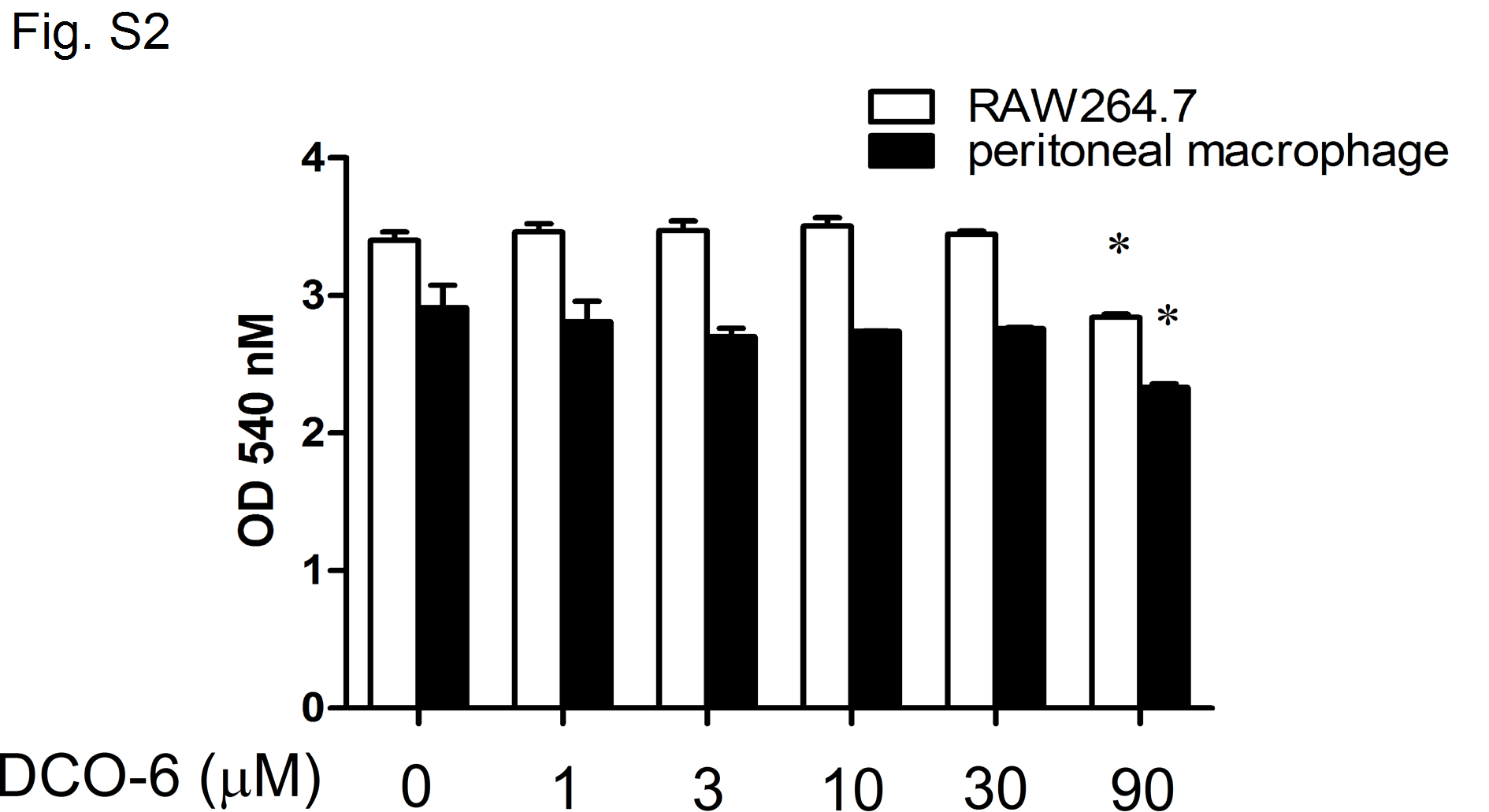

Supplement: Figure S2 — The effect of DCO-6 on cell viability of murine macrophages. RAW264.7 cells or peritoneal macrophages from BALB/c mice were treated with various concentrations of DCO-6. After 24 h of incubation, the cell viability was assessed by MTT assay. Data are shown as means ± S.D. of three independent experiments. *P<0.05 vs medium control. (TIF) [file pone.0037168.s002.tif]

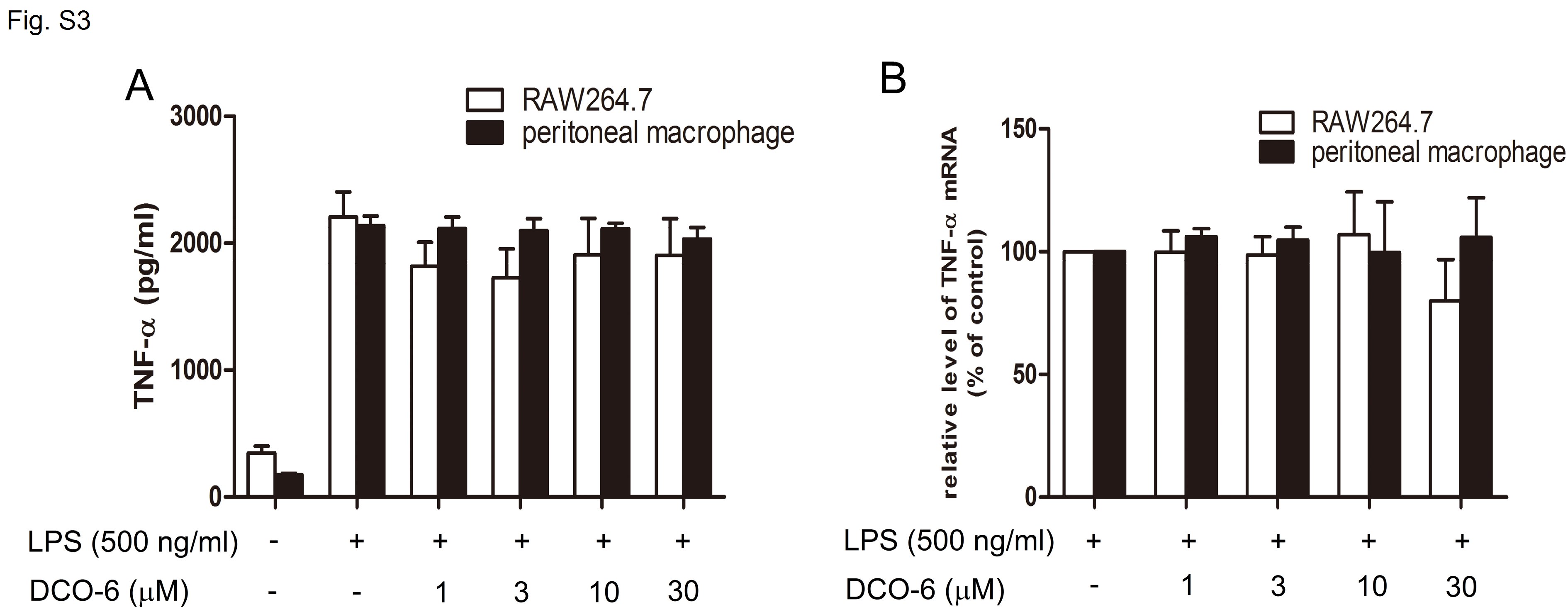

Supplement: Figure S3 — The effect of DCO-6 on TNF-α production in murine macrophages. RAW264.7 cells or peritoneal macrophages from BALB/c mice were treated with various concentrations of DCO-6 in the absence or presence of LPS. (A) The levels of TNF-α in the cell culture medium were determined 24 h after LPS stimulation. (B) The levels of TNF-α mRNA were determined by real-time quantitative PCR 8 h after LPS stimulation. β-actin was used as an invariant control. Data are shown as means ± S.D. of three independent experiments. (TIF) [file pone.0037168.s003.tif]

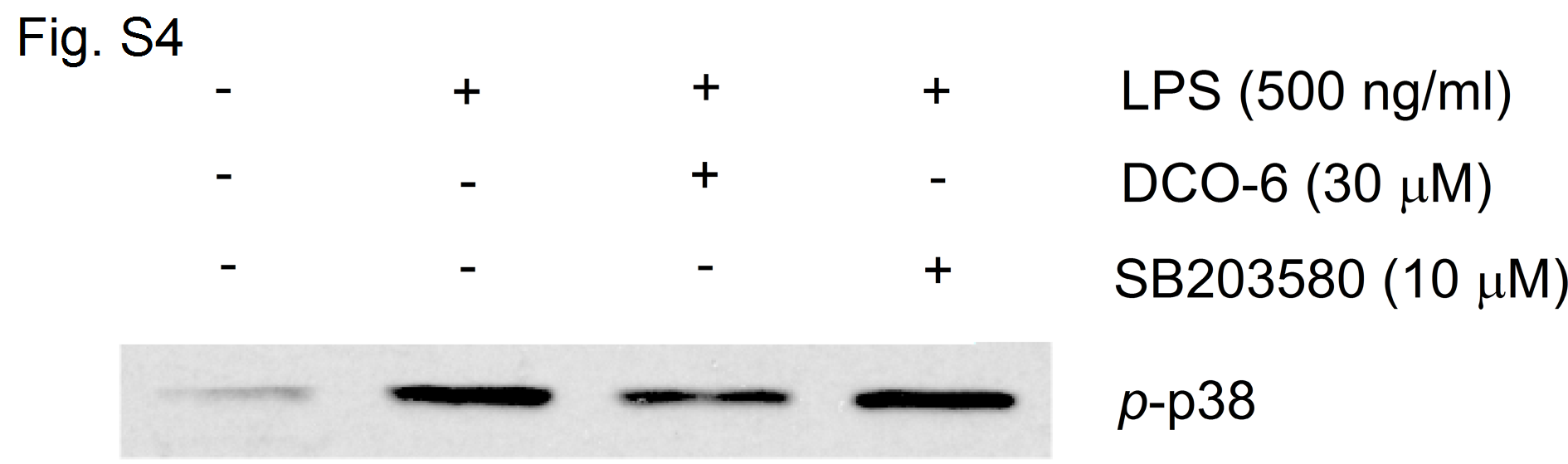

Supplement: Figure S4 — Effects of DCO-6 on LPS-induced p38 MAPK activation in RAW264.7 cells. Cells were treated with DCO-6 and SB203580 in the absence or presence of LPS for 6 h. Endogenous kinases were immunoprecipitated from cell lysates using phospho-p38 (Thr180/Tyr182) antibody bound to protein-A agarose. The phosphorylated p-p38 products were analyzed by immunoblot. Representative data are shown. (TIF) [file pone.0037168.s004.tif]

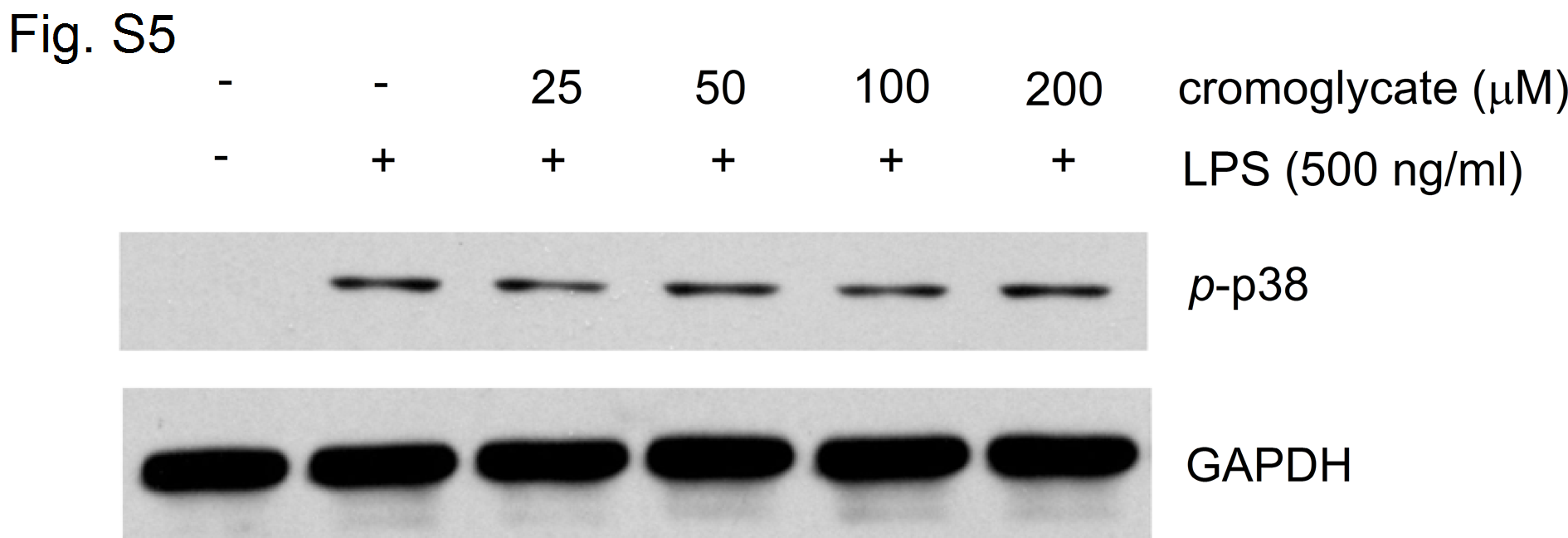

Supplement: Figure S5 — Effects of cromoglycate on p38 MAPK activation induced by LPS in RAW264.7 cells. Cells were treated with various concentrations of cromoglycate in the absence or presence of LPS for 6 h. Whole cell lysates were prepared for Western blotting analysis. The phosphorylated p38 level was analyzed by immunoblot. (TIF) [file pone.0037168.s005.tif]

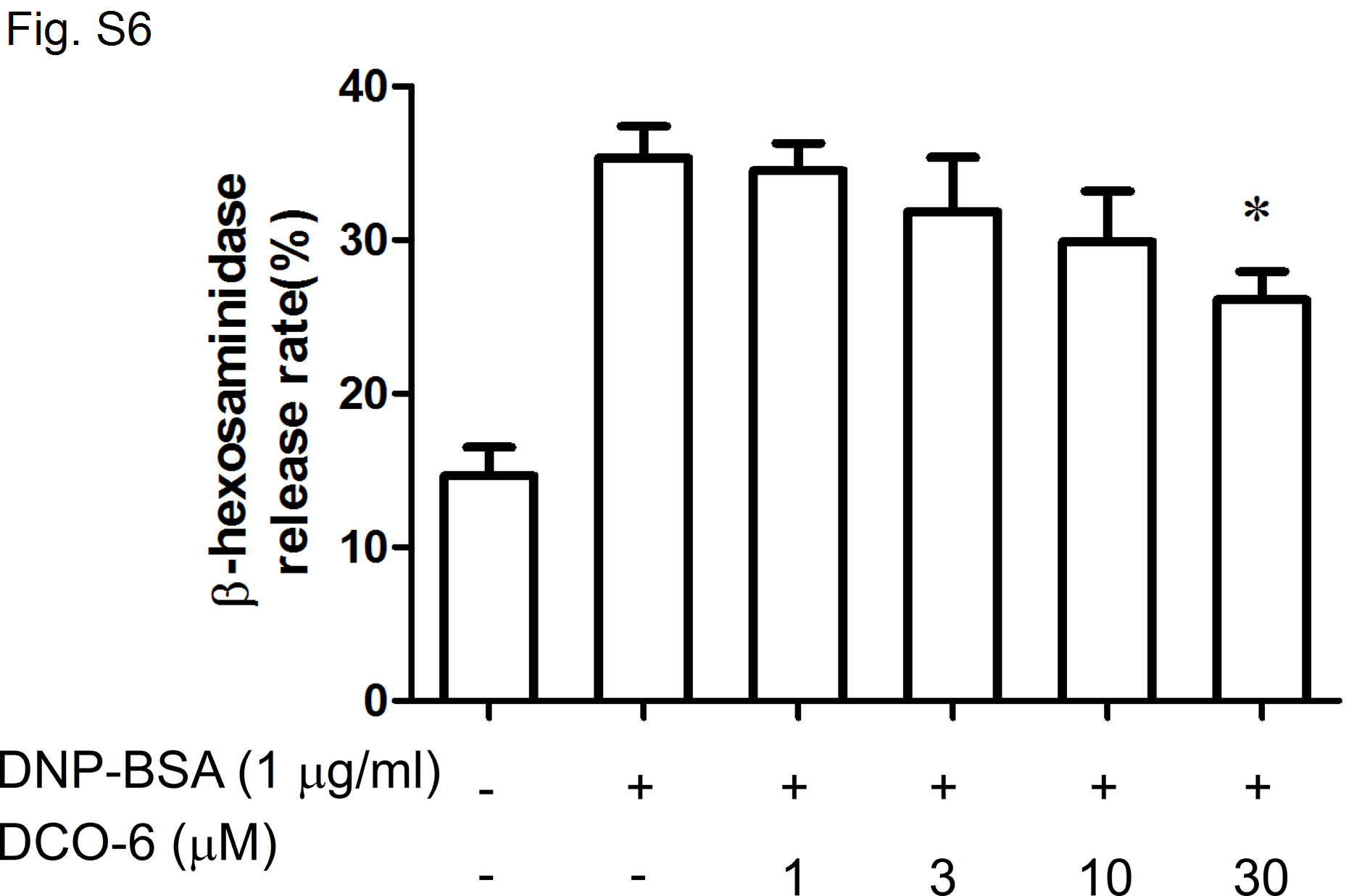

Supplement: Figure S6 — DCO-6 inhibited DNP-BSA-induced β-hexosaminidase release in RBL-2H3 cells. As described in supplementary material S1, cells were seeded in 96-well plates (3×10^4^ cells/well) with or without 0.45 µg/ml anti-dinitrophenyl (DNP) IgE. After overnight incubation, the sensitized cells were treated with the indicated concentration of DCO-6 for 24 h. DNP-BSA (1 µg/ml) were added for 30 min and β-hexosaminidase release levels were detected. *P<0.05 vs DNP-BSA control. (TIF) [file pone.0037168.s006.tif]
