# Supplementary material for: A Novel Chromone Derivative with Anti-Inflammatory Property via Inhibition of ROS-Dependent Activation of TRAF6-ASK1-p38 Pathway
Source: PLoS One. 2012 Jun 15;7(8):e37168. doi: 10.1371/journal.pone.0037168 (PMC3376149; doi:10.1371/journal.pone.0037168)
Supplement: Supplementary Materials S1 — (DOC) [file pone.0037168.s007.doc]

**Supplementary Material**

Chemical Synthesis

5,7-bis(benzyloxy)-4-oxo-4H-chromene-3-carbaldehyde (**1**), was synthesized as previously described [16]. The structure was identified by ^1^H NMR, ^13^C NMR and MS. Melting point was determined on a Taike X-4 digital micromelting point apparatus and uncorrected. The ^1^H NMR and ^13^C NMR measurements were carried out in Bruker DPX-300 spectrometer. ESI-HR-MS were obtained on Esquire 4000 mass spectrometer.

5,7-dihydroxy-4-oxo-4H-chromene-3-carbaldehyde (**2**):

To a solution of **1** (193 mg, 0.5 mmol) in dried CH_2_Cl_2_ (10 ml) at −78 ^o^C was added BBr_3_ (1.1 ml, 1M in CH_2_Cl_2_) dropwise, the reaction solution was stirred at room temperature for 8h. Then the reaction was quenched by addition of H_2_O (3 ml), the afforded solid was filtered, washed with H_2_O (3 ml × 3), dried, and then recrystallized from acetone-CH_2_Cl_2_ to produce the title compound **2**. Yield 94%; mp 296−298 °C; ^1^HNMR (DMSO-d6, 300MHz) *δ*: 6.25 (1H, s), 6.44 (1H, s), 8.78 (1H, s), 10.00 (1H, s), 11.16 (1H, br), 12.35 (1H, br); ^13^C NMR (DMSO-d_6_, 75 MHz) *δ*: 95.08, 100.01, 104.35, 118.61, 157.26, 161.88, 163.80, 165.30, 179.47, 187.26; HRMS-ESI (m/z): calcd for C_10_H_7_O_5_ [M+H]^+^ 207.0293, found: 207.0295.

(E)-5,7-dihydroxy-3-(3-oxo-3-phenylprop-1-en-1-yl)-4H-chromen-4-one **(DCO-6)**:

To a solution of **2** (206 mg, 1.0 mmol) in THF (10 ml) was added Wittig regent and the reaction solution was refluxed for 2 h. TLC indicated that the starting material was consumed completely. The reaction solution was concentrated in vacuo to afford the crude product, which was purified by silica gel column chromatography to give the desired compound DCO-6. Yield 85%; mp 232-234 ^o^C; ^1^H NMR (C_5_D_5_N, 300 MHz) δ: 6.27 (1H, d, J = 1.9 Hz), 6.44 (1H, d, J = 1.9 Hz), 7.54-7.62 (3H, m), 7.69 (1H, t, J = 7.2 Hz), 8.03 (2H, d, J = 7.2 Hz), 8.30 (1H, d, J = 15.7 Hz), 8.93 (1H, s), 11.08 (1H, s), 12.68 (1H, s); ^13^C NMR (C_5_D_5_N, 75 MHz) δ: 94.40, 99.67, 104.12, 117.08, 123.37, 128.26, 128.94, 133.21, 134.90, 137.46, 156.99, 160.63, 162.01, 164.77, 179.95, 189.25; HRMS-ESI (m/z): calcd for C_18_H_13_O_5_ [M+H]^+^: 309.0763 found: 309.0780.

**
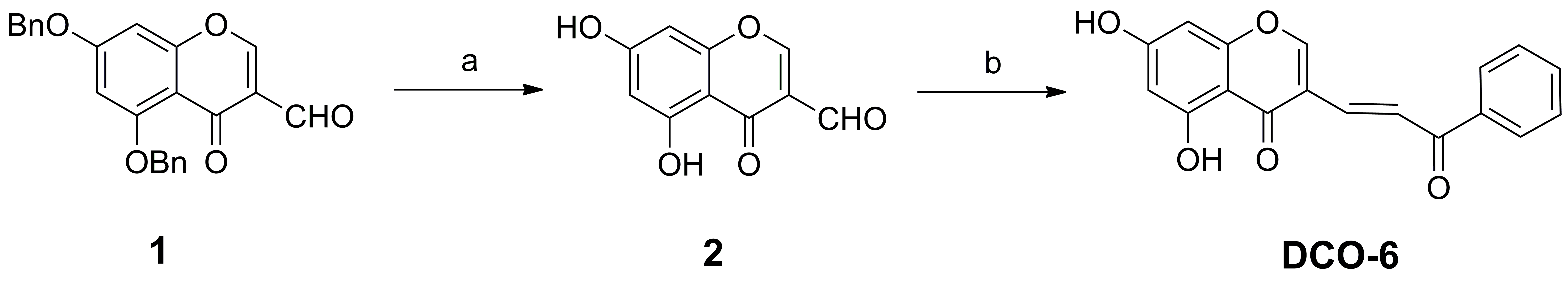
**

Scheme 1. Synthesis of (E)-5,7-dihydroxy-3-(3-oxo-3-phenylprop-1-en-1-yl)-4H-chromen- 4-one (DCO-6)

β-hexosaminidase release assay

Rat basophilic leukemia RBL-2H3 cells were cultured in MEM medium containing 10% FBS and supplemented antibiotics (100 unit/mL penicillin and 100 μg/mL streptomycin) at 37 °C in humidified atmosphere in the presence of 5% CO_2_. Cells were seeded in 96-well plates (3×10^4^cells/well) with or without 0.45 μg/mL anti-dinitrophenyl (DNP) IgE (Sigma Chemical Co, St. Louis, MO, USA). After overnight incubation, the sensitized cells were washed twice with serum-free medium and treated with the indicated concentration of DCO-6 for 24 h. Cells were treated with 1 μg/mL DNP-BSA (Molecular Probes, Eugene, OR, USA) for 30 min and the supernatants and cell lysate were collected. Aliquots (50 μl) of each supernatant and cell lysate were incubated with 50 µL 5 mM *p*-nitrophenyl-N-acetyl-β-D-glucosaminide (Sigma) in 0.1 M citrate buffer (pH 4.5) at 37 °C for 1 h. Enzyme reaction was terminated by adding 100 μl of 0.2 M glycine-NaOH (pH 10.7). The absorbance at 405 nm was measured in a microplate reader. The percentage of β-hexosaminidase released was calculated as follows, β-hexosaminidase activity in supernatant/ (β-hexosaminidase activity in supernatant + β-hexosaminidase activity in cell lysate).
